# The Relationship Between Occupational Standing and Sitting and Incident Heart Disease Over a 12-Year Period in Ontario, Canada

**DOI:** 10.1093/aje/kwx298

**Published:** 2017-08-11

**Authors:** Peter Smith, Huiting Ma, Richard H Glazier, Mahée Gilbert-Ouimet, Cameron Mustard

**Affiliations:** 1 Institute for Work and Health, Toronto, Ontario, Canada; 2 Department of Epidemiology and Preventive Medicine, Monash University, Melbourne, Australia; 3 Dalla Lana School of Public Health, University of Toronto, Toronto, Ontario, Canada; 4 Institute for Clinical Evaluative Sciences, Toronto, Ontario, Canada; 5 Department of Family and Community Medicine, University of Toronto and St. Michael’s Hospital, Toronto, Ontario, Canada; 6 Centre for Urban Health Solutions, Li Ka Shing Knowledge Institute, St. Michael’s Hospital, Toronto, Ontario, Canada; 7 Centre de Recherche du CHU de Québec, Hôpital du Saint-Sacrement, Quebec, Canada

**Keywords:** administrative data, Canada, heart diseases, occupational exposure, sitting position, standing position

## Abstract

While a growing body of research is examining the impacts of prolonged occupational sitting on cardiovascular and other health risk factors, relatively little work has examined the effects of occupational standing. The objectives of this paper were to examine the relationship between occupations that require predominantly sitting and those that require predominantly standing and incident heart disease. A prospective cohort study combining responses to a population health survey with administrative health-care records, linked at the individual level, was conducted in Ontario, Canada. The sample included 7,320 employed labor-market participants (50% male) working 15 hours a week or more and free of heart disease at baseline. Incident heart disease was assessed using administrative records over an approximately 12-year follow-up period (2003–2015). Models adjusted for a wide range of potential confounding factors. Occupations involving predominantly standing were associated with an approximately 2-fold risk of heart disease compared with occupations involving predominantly sitting. This association was robust to adjustment for other health, sociodemographic, and work variables. Cardiovascular risk associated with occupations that involve combinations of sitting, standing, and walking differed for men and women, with these occupations associated with lower cardiovascular risk estimates among men but elevated risk estimates among women.

Cardiovascular disease continues to be a leading cause of morbidity and mortality worldwide, in particular in high-income countries (1). Sedentary behavior is gaining increasing attention as a modifiable risk factor for a number of chronic disease outcomes, including cardiovascular disease (2, 3). While sedentary behavior in general has been associated with increased risk of multiple disease outcomes, the available evidence that sedentary occupational activity is a cardiovascular risk factor is less convincing (3). A pooled analysis of 5 cohorts from England and 2 cohorts from Scotland (total *n* = 5,214) reported no relationship between prolonged occupational sitting, compared with occupations involving standing and walking about, in relation to cardiovascular mortality over a 12.9-year follow-up period (4). Another recent examination of the relationship between occupational sitting time and ischemic heart disease among a Danish cohort of over 2,500 men and women also reported no relationship between sitting time and ischemic heart disease over a 12-year follow-up (5).

Compared with the research on prolonged sitting, relatively little research has examined the health effects of prolonged occupational standing (6–8). Although few in number, studies have demonstrated a relationship between prolonged standing at work and various cardiovascular outcomes (9–12) as well as other health outcomes such as musculoskeletal pain (7, 8). The potential mechanisms linking prolonged standing to cardiovascular outcomes include blood pooling in the lower limbs, increased hydrostatic venous pressure, and enhanced oxidative stress (6, 10, 13). Despite these findings, there has been a greater degree of research emphasis on understanding the feasibility and effectiveness of reducing prolonged sitting as opposed to prolonged standing (6, 14).

The objectives of this study were to examine the relationship between imputed occupational body position exposures focused on sitting, standing, walking, and other body positions, and incident heart disease over a 12-year period in Ontario, Canada.

## METHODS

The data source for this analysis was respondents to the 2003 Canadian Community Health Survey (CCHS), whose responses were linked to administrative information in the Ontario Health Insurance Plan database covering physician services and the Canadian Institute for Health Information Discharge Abstract Database for hospital admissions. Information from the Ontario Health Insurance Plan database and the Canadian Institute for Health Information Discharge Abstract Database was available up to March 31, 2015. The administrative databases were linked to the CCHS responses using unique, encoded identifiers and analyzed at the Institute for Clinical Evaluative Sciences. The accuracy of the linkage was verified against the Ontario Registered Persons Database using personal information provided by respondents, such as health number, given name and surname, date of birth, age, sex, and postal code.

The CCHS collects information on health conditions, health behaviors, and working conditions from representative cross-sectional samples of the Canadian population. The overall response rate from respondents from Ontario to the 2003 CCHS was 78.5% (15). Of the 40,507 Ontario respondents to the 2003 CCHS, 34,950 (86%) gave permission for their survey responses to be linked to administrative health-care data. Of this sample, a successful linkage was obtained for 33,679 respondents (96% of those who gave permission to link). For the purpose of this analysis we focused on currently employed respondents working more than 15 hours per week, who were 35–74 years of age (*n* = 8,873). Ethics approval for secondary data analysis was granted through the University of Toronto, Health Sciences Ethics Board, and the Research Ethics Board of Sunnybrook Health Sciences Centre.

### Patient involvement

This study was an analysis of secondary survey and administrative data. As such, patients were not involved.

### Outcome: incident heart disease

Incident heart disease over the follow-up period was derived using the Ontario Myocardial Infarction Database and the Ontario Congestive Heart Failure Database. Both of these databases were developed using validated algorithms, with sensitivity and specificity estimates of approximately 0.85 or higher (16–18). These databases capture cases of heart disease from 1992 onward, providing an approximate 12-year look-back window for prevalent cases of each condition in our sample. In all regression models, respondents were right censored at the development of heart disease, death from causes other than heart disease, or the end of the follow-up period (March 31, 2015).

### Main independent variable: primary type of occupational posture or body movement

The primary type of posture or body movement required to perform each respondent’s occupation was imputed based on occupational information from the Human Resources and Skills Development Canada’s Career Handbook (19). The Career Handbook assigns various occupational exposures to occupations at the 4-digit occupational level, equating to 520 different job titles. For each occupational title, minimum and maximum exposures for multiple dimensions of work were assigned by trained occupational analysts using a modified Delphi procedure. After the consensus ratings for each occupation and exposure had been developed, the occupations were additionally reviewed by task to identify potential abnormalities (19).

The primary type of posture or body movement for each occupational title involves one of 4 possible categories: occupations requiring primarily sitting; occupations involving primarily standing and/or walking; occupations involving combinations of sitting, standing, and walking; and work that involves body postures other than sitting, standing, and walking, such as bending, stooping, kneeling, and crouching. Using the minimum and maximum occupational exposures, we were able to classify occupations into those that require predominantly sitting (where minimum and maximum body position was sitting); those that require predominantly standing (where minimum and maximum body position was standing); occupations with opportunities for sitting, standing, and walking; and occupations that predominantly involve working in other body positions. Examples of the most common types of occupations for men and women within each of these groups are provided in Web Table 1 (available at https://academic.oup.com/aje).

### Sociodemographic and health-related covariates

Other measures included in analyses were age; whether the respondent was male or female; marital status and presence of children under 12 in the house; highest educational level obtained; whether the respondent was born in Canada; respondent ethnicity (“white” versus other categories); living location (urban/rural); and self-reported chronic medical conditions that have been diagnosed by a health professional and are expected to last, or have lasted, more than 6 months. For chronic health conditions the following groups were derived: diabetes, high blood pressure, back problems, mood and anxiety disorders, and other chronic conditions. We also included a measure of whether a long-term physical or mental health condition limited the type or amount of activity the respondent could do at work (never, sometimes, or often).

### Other work-related exposures

In addition to occupational posture and body movement, a variety of other occupational exposures were also included. Self-reported exposures included the usual hours worked by the respondent each week (continuous), the number of weeks worked in the previous 12 months (weeks worked: 1–26, 27–49, 50 or more weeks), and current shift schedule (regular, evening or night shift, rotating, or other shift schedules). Imputed occupational exposures based on occupational title included the handling of loads 10 kg or greater; exposure to dangerous chemical substances; exposure to constant or intermittent noise likely to cause distraction or possible hearing loss; exposure to oscillating or quivering motions (vibration); and exposure to noxious, intense, or prolonged odors. Imputed exposures were defined as dichotomous variables.

### Body mass index and health behaviors

The following measures were available in the data set: body mass index (BMI) based on self-reported height and weight (underweight/normal weight, overweight, obese); current smoking status (regular smoker, occasional smoker, nonsmoker); alcohol consumption (nondrinker, regular drinker but never having 5 or more drinks in one sitting, regular drinker who has 5 or more drinks on an occasional to weekly basis); and leisure-time physical activity categorized according to Statistics Canada’s definitions (inactive = less than 1.5 kcal/kg/day (e.g., walking less than half an hour each day), moderately active = between 1.5 and 2.9 kcal/kg/day (e.g., walking 30–60 minutes a day, or taking an hour-long exercise class 3 times a week), active = at least 3 kcal/kg/day (e.g., walking an hour a day or jogging 20 minutes a day)). The handling of BMI and health behaviors in the analytical models is described in further detail below.

### Analyses

Of the original sample of 8,873 respondents, 350 (4%) either reported having preexisting heart disease or were captured in the Ontario Myocardial Infarction Database or the Ontario Congestive Heart Failure Database prior to the interview date, and were removed, leaving a sample of 8,523 respondents. Of this sample, 562 respondents were missing information on work exposures, with an additional 641 respondents missing information on sociodemographic characteristics, health measures, or health behaviors, leaving a final analytical sample of 7,320 respondents (50% male), which is 86% of the eligible sample. A logistic regression analysis examined variables associated with the probability of missing work exposures, and missing sociodemographic, health, or health-behavior measures. Men were more likely than women to be missing work-exposure information, while women, respondents in urban locations, and those working in other body positions were more likely to be missing sociodemographic, health, or health-behavior measures. No relationship was found between age and having missing information on work exposures or having missing information on sociodemographic, health, or health-behavior measures.

Initial descriptive analyses examined the relationships between incident heart disease and occupational standing and sitting. Time-to-event regression models then examined the relationships between occupational standing and sitting exposures using a series of nested regression models. The first model was adjusted for age, sex, education, and weeks of work in the previous 12 months (minimal-adjustment model). Additional adjustment was made for other sociodemographic variables, followed by a model that additionally included other work exposures. A final model adjusted for BMI and health behaviors (smoking, alcohol consumption, leisure-time physical activity). The reason for the separate adjustment for BMI and health behaviors is because it is not clear whether these factors are confounders or mediators in the occupational-exposure-to-heart-disease outcome relationship. Because it is unlikely that an individual’s BMI or health behaviors result in them being in particular occupations, including these factors in regression models could be considered a form of overadjustment (20).

To ensure an adequate number of predictors to events in our final models, work exposures that were not related to heart disease in either univariate or multivariable models were removed. The main exclusions were other occupational exposures, which included dangerous chemical substances, noise, vibration, and odors. Regression models were fitted for the full sample and separately for men and women to examine differences in the relationships between occupational exposures and heart disease outcomes among men and women. Differences between estimates from sex-specific regression models were assessed using methods that take into account the estimate and standard error around the estimate from stratified regression models (21, 22). A final set of models examined the relationships between occupational exposures and incident heart disease, removing events that occurred in the first 2 years of follow-up to reduce the possibility of reverse causation.

All analyses were conducted in SAS, version 9.4 (SAS Institute, Inc., Cary, North Carolina), and survey weights were applied to the sample to account for the initial probability of selection into the CCHS and nonresponse to the survey, as recommended by Statistics Canada (15). To account for the clustered design of the CCHS, variance estimates around each prevalence and hazard ratio have been adjusted using 500 bootstrap replicate weights using the SURVEY procedures in SAS (SAS Institute, Inc.) (23).

## RESULTS

Table 1 presents descriptive information for incident heart disease across our main independent variable and for men and women. Over the study period, there were 83,424 person-years of follow-up (median follow-up 11.73 years); 3.4% of the study population developed heart disease, with a higher incidence among men (4.6%) than women (2.1%). The risk of incident heart disease was elevated among occupations requiring predominantly standing compared with occupations requiring predominantly sitting. No statistically significant differences were observed in the incidence of heart disease across other occupational exposure groups. Additional information on the incidence of heart disease across all other study variables is available in Web Table 2.

**Table 1.** Incidence of Heart Disease Over a 12-Year Period Across Occupational Standing and Sitting Exposures in a Cohort of Employed Workers Aged 35–74 Years (*n* = 7,320), Ontario, Canada, 2003–2015

Variable	No. of Workers	All (*n* = 7,320)		Test for Difference	
		Heart Disease Incidence^a^	95% CI^b^	Wald χ^2^	*P* Value
Sex					
Men	3,828	4.62	3.62, 5.61	Referent	
Women	3,492	2.08	1.27, 2.89	15.12	<0.001
Primary type of body posture or movement					
Sitting	2,683	2.82	2.04, 3.60	Referent	
Standing	682	6.59	3.21, 9.97	4.56	0.03
Sitting, standing, and walking	2,429	2.79	1.63, 3.94	0.00	0.97
Other body positions	1,526	4.01	2.82, 5.20	2.71	0.10

Abbreviation: CI, confidence intervals

^a^ All estimates were weighted for the probability of selection into the Canadian Community Health Survey (CCHS) and initial survey nonresponse.

^b^ Confidence limits have been adjusted to take into account the clustered design of the Canadian Community Health Survey.

Table 2 presents hazard ratios for occupational standing and sitting after adjustment for age, sex, education, and weeks worked (model 1); model 1 with other sociodemographic and health-related conditions (model 2); model 2 with other work exposures (model 3); and model 3 with health behaviors and BMI (model 4). Similar to the descriptive analyses, predominantly standing occupations were associated with an increased risk of heart disease compared with sitting occupations (hazard ratio (HR) = 2.18, 95% confidence interval (CI): 1.11, 4.27) after adjustment for sociodemographic, health, and work-related variables. Additional adjustment for BMI and health behaviors attenuated this association slightly; however, workers employed in occupations that required predominantly standing were still almost twice as likely to have incident heart disease over the study period compared with those whose work predominantly required sitting (HR = 1.97, 95% CI: 0.99, 3.90). These estimates remained robust to potential reverse causation, slightly strengthening, and in the case of model 4 attaining statistical significance, after the removal of incident cases of heart disease that occurred in the first 2 years of follow-up (for model 3: HR = 2.21, 95% CI: 1.10, 4.46; for model 4, HR = 2.06, 95% CI: 1.00, 4.24; results not shown).

**Table 2.** Hazard Ratios Over a 12-Year Period for Sitting and Standing Occupational Exposures and Incident Heart Disease Among Employed Canadian Workers Aged 35–74 Years (*n* = 7,320), Ontario, Canada, 2003–2015

Primary Type of Body Posture or Movement	Model 1^a^		Model 2^b^		Model 3^c^		Model 4^d^	
	HR	95% CI	HR	95% CI	HR	95% CI	HR	95% CI
Sitting	1.00	Referent	1.00	Referent	1.00	Referent	1.00	Referent
Standing	2.32^e^	1.16^e^, 4.62^e^	2.28^e^	1.16^e^, 4.45^e^	2.18^e^	1.11^e^, 4.27^e^	1.97	0.99, 3.90
Sitting, standing, and walking	0.97	0.58, 1.61	0.93	0.56, 1.55	0.93	0.56, 1.54	0.97	0.58, 1.62
Other body positions	1.09	0.70, 1.69	1.04	0.66, 1.66	1.04	0.42, 2.57	1.07	0.43, 2.65

Abbreviations: CI, confidence intervals; HR, hazard ratio.

^a^ Model 1 adjusted for age, sex, weeks worked in the previous 12 months, and highest level of education.

^b^ Model 2: model 1 with additional adjustment for immigrant status, ethnicity, marital status, presence of children, activity restrictions at work, diabetes, hypertension, arthritis, mood and anxiety disorders, and other chronic conditions.

^c^ Model 3: model 2 with additional adjustment for shift work and physical work demands.

^d^ Model 4: model 3 with additional adjustment for smoking, leisure-time physical activity, alcohol consumption, and body mass index.

^e^ Estimates with statistically significant relationships with heart disease.

Table 3 provides hazard ratio estimates for occupational standing and sitting exposures separately for men and women. The hazard ratio estimates for standing occupations were relatively consistent among men and women (HR = 2.01 for men; HR = 1.86 for women). However, the hazard ratio estimates for occupations that require combinations of sitting, standing, and walking, compared with those that required predominantly sitting, differed between men and women. Among men, occupations involving sitting, standing, and walking were associated with a protective hazard ratio for heart disease risk (HR = 0.61, 95% CI: 0.33, 1.13), but an elevated hazard ratio was observed among women (HR = 1.80, 95% CI: 0.78, 4.12). While neither of these estimates reached statistical significance, the difference between the hazard ratio estimates for men and women was statistically significant. Similar to the hazard ratio estimates in the full sample, removing incident cases of heart disease in the first 2 years of follow-up strengthened these estimates, with the protective hazard ratio estimate for combinations of standing, walking and sitting achieving statistical significance among men in this sensitivity analysis (HR = 0.49, 95% CI: 0.27, 0.88; results not shown).

**Table 3.** Hazard Ratios Over a 12-Year Period for Sitting and Standing Occupational Exposures and Incident Heart Disease Among Employed Men and Women Aged 35–74 Years (*n* = 7,320)^a^, Ontario, Canada, 2003–2015

Primary Type of Body Posture or Movement	Men		Women		χ^2^ for Difference^b^	*P* for Difference^b^
	HR	95% CI	HR	95% CI		
Sitting	1.00	Referent	1.00	Referent		
Standing	2.01	0.85, 4.71	1.86	0.45, 7.71	0.01	0.93
Sitting, standing, and walking	0.61	0.33, 1.13	1.80	0.78, 4.12	4.22^c^	0.04^c^
Other body positions	0.93	0.33, 2.64	0.68	0.16, 2.96	0.11	0.74

Abbreviations: CI, confidence intervals; HR, hazard ratio.

^a^ The model adjusted for age, weeks worked in the previous 12 months, highest level of education, immigrant status, ethnicity, marital status, presence of children, activity restrictions at work, diabetes, hypertension, arthritis, mood and anxiety disorders, other chronic conditions, shift work, and physical work demands, corresponding to Model 3 in Table 2.

^b^ χ^2^ and *P* values are for differences in estimates for men compared with women.

^c^ Estimates were significantly different for men and women.

## DISCUSSION

The health impact of increases in sedentary occupational exposures has attracted a great deal of recent research interest (2, 3, 14). While work in this area has examined the impact of prolonged occupational sitting, relatively little research has examined the health impacts of prolonged standing. In this study of more than 7,300 labor-market participants in Ontario, Canada, we observed that occupations that involve predominantly standing were associated with a 2-fold risk of incident heart disease, compared with predominantly sitting occupations, over a 12-year follow-up period. Estimates were similar for men and women and robust to adjustment for other health, sociodemographic, and work exposures. Adjustment for health behaviors and body mass index led to an attenuation of hazard ratio estimates associated with predominantly standing occupations. After this adjustment, although the resulting hazard ratio was still close to 2, the lower bound of the confidence interval was just below 1. In addition, the risk associated with occupational standing strengthened in a sensitivity analysis in which heart cases in the first 2 years of follow-up were removed. The cardiovascular risk associated with occupations that involve combinations of sitting, standing, and walking differed for men and women, with these occupations associated with protective cardiovascular risk estimates among men but elevated (although not statistically significant) cardiovascular risk estimates among women. Taken together these findings suggest that occupational standing should receive similar, if not more, attention than occupational sitting, in relation to potential adverse cardiovascular outcomes (6, 14).

These findings have important implications for the prevention of cardiovascular disease and the role of the work environment as a cardiovascular risk factor. First, predominantly standing occupations, as opposed to predominantly sitting occupations, comprised the occupational body position category most strongly associated with heart disease. This finding suggests that combinations of sitting and standing are likely to have beneficial cardiovascular health benefits. However, the introduction of this type of work environment should not focus only on occupations that involve prolonged sitting but also on occupations that involve prolonged standing (14). Predominantly standing occupations were not as common as predominantly sitting occupations in our sample. The prevalence of this exposure was just under 10% among our cohort of employed respondents, who were aged 35 or older and free of heart disease at baseline.

Second, the sex-specific differences in the hazard ratio estimates of standing, sitting, and walking occupations demonstrate that other work-context factors can also shape the effectiveness of workplace-based interventions to increase occupational physical activity. While occupations that involved combinations of sitting, standing, and walking were associated with a decreased risk of heart disease among men, they were associated with an increased risk of heart disease among women. Examination of common occupational titles within sitting, standing, and walking occupations showed that the types of occupations within this occupational group differed greatly for men and women (Web Table 1), and this is one potential reason for this difference in estimates (6, 24). In addition, research has shown that the physical and psychosocial work environment can differ for men and women even within the same occupational title (25), and it is possible that these differences are more pronounced in sitting, standing, and walking occupations than they are in prolonged standing or prolonged sitting occupations. This differential association of occupations that involve greater walking and cardiovascular outcomes for men and women suggests that a focus solely on occupational activity, ignoring other occupational conditions such as the psychosocial and other aspects of the physical work environment, is unlikely to lead to meaningful changes in cardiovascular risk (26).

These findings should be interpreted in light of the following strengths and limitations. The administrative data used to capture heart disease require use of health services, and therefore they will not capture out-of-hospital myocardial infarction or angina. However, because of the publically funded health-care system in Ontario, out-of-hospital services are likely relatively rare in comparison with those captured in each database. The assessment of working conditions involved assessment at one point in time, and we have no information beyond the 12 months prior to the survey as to the occupational title, or labor-market participation, of each respondent. While the use of imputation based on occupational title does have advantages in limiting the potential for common-method bias to inflate the association between perceived occupational standing or sitting and unmeasured risk factors for heart disease (27), as well as the demonstrated inconsistency between self-reported and objectively measured sitting (28), it also presents a limitation in that potentially important differences within occupational groups (e.g., the ability to take breaks during the work schedule) are assumed to be equivalent across all members of the same occupational classification, although this may not be the case (6). In addition, we have limited information on the amount of time spent standing or sitting in each occupational group, and this may be important in assessing the relationship between sitting and standing and health outcomes (29). The potential misclassification introduced by these factors would likely lead to a bias toward the null in the estimates for occupational sitting and standing, and therefore the estimates presented in this paper may be underestimates of the true association between occupational standing and cardiovascular disease.

Strengths of the present study include our ability to adjust for a wide range of covariates, related and unrelated to work, that could potentially confound the relationship between occupational body position and incident heart disease, and our ability to examine the potential for reverse causation in explaining our findings. Our ability to minimize reverse causation could be one explanation for why our findings differ from previous studies that have demonstrated a protective relationship between greater daily time spent standing (not necessarily occupational) and mortality (30, 31).

In conclusion, in a study of more than 7,300 Canadians, occupations that involve primarily standing represent an important, but often overlooked, cardiovascular risk factor, one that is independent of other health, sociodemographic, and labor-market characteristics. This evidence suggests that primary prevention efforts targeted toward reducing occupational standing should be considered, while taking into account the broader occupational context and potential differences in occupational context between men and women.

## Supplementary Material

Web Material

## Abbreviations

BMI: body mass index
CCHS: Canadian Community Health Survey
CI: confidence interval
HR: hazard ratio
