# Supplementary material for: The Relationship Between Occupational Standing and Sitting and Incident Heart Disease Over a 12-Year Period in Ontario, Canada
Source: Am J Epidemiol. 2017 Aug 11;187(1):27–33. doi: 10.1093/aje/kwx298 (PMC5860480; doi:10.1093/aje/kwx298)
Supplement: Web Material [file kwx298smithwebmaterialfinal.pdf]

Web Material

Web Table 1. Common occupational titles worked by men and women within each body position category

Sitting		Standing		Sitting, Standing, Walking		Other body positions	
**Men**	**Women**	**Men**	**Women**	**Men**	**Women**	**Men**	**Women**
Truck Drivers	Administrative Officers	Retail Salespersons and Sales Clerks	Retail Salespersons and Sales Clerks	Motor Vehicle Assemblers, Inspectors and Testers	Registered Nurses	Automotive Service Technicians, Truck and Bus Mechanics and Mechanical Repairers	Nurse Aides, Orderlies and Patient Service Associates
Administrative Officers	Secretaries (Except Legal and Medical)	Cooks	Cooks	Delivery and Courier Service Drivers	Elementary School and Kindergarten Teachers	Shippers and Receivers	Light Duty Cleaners
Financial Auditors and Accountants	Administrative Clerks	Other Metal Products Machine Operators	Food and Beverage Servers	Retail Trade Managers	Cashiers	Janitors, Caretakers and Building Superintendents	Janitors, Caretakers and Building Superintendents
Professional Occupations in Business Services to Management	Accounting and Related Clerks	Machining Tool Operators	Customer Service Representatives - Financial Services	Sales Representatives - Wholesale Trade	Elementary and Secondary School Teacher Assistants	Material Handlers	Shippers and Receivers
		Plastics Processing Machine Operators	Other Metal Products Machine Operators				

Web Table 2. Incidence of Heart Disease Over a 12-Year Period across all study variables in a Cohort of Employed Canadian Workers Aged 35 to 74 Years of Age (N = 7,320)

	All (N = 7,320)			Test for difference	
	**N**	**Heart disease incidence**	**95% CI**	**Wald chi-square**	**P-value**
**Age Group**					
35 to 44 years	3,505	1.80	1.17, 2.43	ref	
45 to 54 years	2,641	3.95	2.73, 5.17	9.50	0.002
55+ years	1,174	6.97	5.02, 8.93	24.43	< 0.001
**Weeks Worked**					
1 to 26 weeks	228	8.86	0.42, 17.30	1.63	0.20
27 to 49 weeks	706	2.13	1.25, 3.01	4.88	0.03
50+ weeks	6,385	3.35	2.72, 3.99	ref	
**Usual Work Hours**					
15 to 34 hours	1,004	3.33	1.75, 4.92	0.01	0.94
35 to 40 hours	3,253	3.26	2.22, 4.30	ref	
41 to 49 hours	1,275	2.86	1.77, 3.95	0.27	0.60
50+ hours	1,787	4.10	2.60, 5.61	0.81	0.37
**Highest Education Completed**					
Less than secondary	736	6.20	3.93, 8.47	ref	
Secondary Schooling	2,040	3.43	2.22, 4.63	4.48	0.03
Post-secondary (below Bachelors)	2,795	3.24	2.10, 4.39	5.22	0.02
Bachelors and higher	1,749	2.46	1.33, 3.59	8.39	0.004
**Canadian Born**					
Yes	5,138	3.25	2.59, 3.90	ref	
No	2,182	3.78	2.31, 5.24	0.42	0.52
**Ethnicity**					
White	6,285	3.44	2.78, 4.09	ref	
Non-White	1,035	3.22	0.87, 5.57	0.03	0.86
**Geographic Location**					
Urban	6,190	3.46	2.72, 4.20	ref	
Rural	1,130	3.10	2.07, 4.13	0.31	0.58
**Marital Status**					
Married/Common-Law	5,882	3.18	2.51, 3.85	ref	
Divorced/Separated/Widowed	843	4.35	1.96, 6.75	0.85	0.36
Single	595	4.30	2.26, 6.33	1.05	0.30

Web Table 2 (cont). Incidence of Heart Disease Over a 12-Year Period across all study variables in a Cohort of Employed Canadian Workers Aged 35 to 74 Years of Age (N = 7,320)

	All (N = 7,320)			Test for difference	
	**N**	**Heart disease incidence**	**95% CI**	**Wald chi-square**	**P-value**
**Children Less than 12 in household**					
Yes	2,395	2.38	1.29, 3.46	ref	
No	4,925	3.91	3.10, 4.71	4.93	0.03
**Often has restrictions in activity at work due to health condition**					
Yes	277	7.65	2.52, 12.79	2.81	0.09
No	7,043	3.24	2.60, 3.88	ref	
**Hypertension**					
Yes	929	4.11	2.51, 5.72	0.84	0.36
No	6,391	3.30	2.62, 3.99	ref	
**Diabetes**					
Yes	263	10.36	4.75, 15.98	6.29	0.01
No	7,057	3.15	2.52, 3.77	ref	
**Arthritis**					
Yes	1,082	4.21	2.53, 5.89	1.03	0.31
No	6,238	3.27	2.56, 3.97	ref	
**Mood and Anxiety**					
Yes	522	1.48	0.57, 2.39	12.60	< 0.001
No	6,798	3.55	2.86, 4.24	ref	
**Other Chronic Conditions**					
Yes	3,533	3.72	2.67, 4.76	0.87	0.35
No	3,787	3.11	2.37, 3.86	ref	
**Current shift schedule**					
Regular	5,583	3.15	2.39, 3.90		
Evening or night	455	2.71	1.10, 4.31	0.24	0.63
Rotating	777	4.68	2.50, 6.87	1.69	0.19
Other shift schedules	505	4.94	2.91, 6.98	2.63	0.11
**Handling of loads**					
Limited and Light	5,637	3.17	2.44, 3.90		
Medium and Heavy	1,683	4.21	3.00, 5.41	2.06	0.15

Web Table 2 (cont). Incidence of Heart Disease Over a 12-Year Period across all study variables in a Cohort of Employed Canadian Workers Aged 35 to 74 Years of Age (N = 7,320)

	All (N = 7,320)			Test for difference	
	**N**	**Heart disease incidence**	**95% CI**	**Wald chi-square**	**P-value**
**Smoking**					
Never smoker	2,246	2.67	1.24, 4.11	ref	
Former smoker	3,156	2.52	1.90, 3.14	0.04	0.85
Occasional smoker	356	5.45	2.40, 8.50	2.63	0.11
Daily smoker	1,562	5.78	3.97, 7.60	6.97	0.01
**Leisure time physical activity**					
Active	1,603	4.10	2.60, 5.61	ref	
Moderately Active	1,917	2.46	1.20, 3.71	2.71	0.10
Inactive	3,799	3.59	2.78, 4.40	0.35	0.56
**Alcohol Consumption**					
Non-Drinker	1,042	4.73	2.79, 6.67	3.14	0.08
Non-Binge Drinker	3,448	2.81	1.93, 3.69	ref	
Binge drinker (last 12 months)	2,829	3.64	2.61, 4.68	1.45	0.23
**Body Mass Index**					
Normal weight (includes underweight)	3,337	2.65	1.84, 3.47	ref	
Overweight	2,751	2.74	2.01, 3.47	0.03	0.87
Obese	1,232	6.93	4.46, 9.40	10.44	0.001

CI – confidence interval

All estimates are weighted for the probability of selection into the CCHS and initial survey non-response. Confidence limits have been adjusted to take into account the clustered design of the CCHS. Although age-group, weeks worked and work hours are included in the table as categorical variables they were included in regression models as continuous measures
